# Application of Functional Hazard Analysis Technique (FuHA) in the risk assessment and accident management: a case study in a textile industry

**Published:** 2019-08

**Authors:** Mohammad Khandan, Alireza Koohpaei, Zeinab Hosseinzadeh, Abbas Sadeghi

**Affiliations:** ^*a*^Qom University of Medical Sciences, Qom, Iran.; ^*b*^Shahid Beheshti University of Medical Sciences, Tehran, Iran.

## Abstract

**Background:**

Nowadays, accompanied with the rapid advances in industries and technology, many concerns about the implications of technological development are threatening human life. High application of pressure vessels and their high operational risks indicate the need to identify the hazards and causes assessment of possible incidents. This study was conducted to evaluation of the risk of boiler unit in a textile industry in Qom and in 2015.

**Methods:**

In this study, the operational hazards of a boiler unit were analyzed using Functional Hazard Analysis technique (FuHA). FuHA is one of the system risk analysis methods and is considered as a qualitative technique. This method identifies and analyzes the risks of the system. In fact, the purpose of this technique is to determine the system s risks through functional analysis.

**Results:**

Based on the application of FuHA technique on the selected boiler unit, 26 cases of hazard and functional failure status were identified. From this identified failure 3 cases were unacceptable, 11 cases were undesirable, 10 cases were acceptable but needed revision and 2 cases were evaluated as negligible risk finally. In total, for identified hazards, 49 causative factors were identified and determined. Among the identified hazards, failure to perform or inaccurate operation of the boiler valve with a 2A risk index (unacceptable) was identified as the most critical risk in the studied system. This situation can lead to an increase in Boiler pressure, then blasting and financial losses or injuries to individuals and properties.

**Conclusions:**

The obtained results showed that the FuHA technique has a desirable capability to identify and analyze the system and subsystems systemic risk especially the software subsystems. Therefore, this method can be used as one of the strategies for assessing hazards in industrial environments.

**Keywords:**

Safety, Boiler, Risk assessment, Functional hazard analysis, Textile industry

